# A Biliary Endoprosthesis Functioning After Six Years

**DOI:** 10.1155/DTE.1.113

**Published:** 1994

**Authors:** Alan Plumer, K. Iswara, Jerome H. Siegel

**Affiliations:** 1 Department of Medicine Division of Gastroenterology Maimonides Medical Center Brooklyn NY USA; 2 Maimonides Medical Center Brooklyn NY USA; 3 Beth Israel Medical Center North Division New York NY USA; 4 Dept. of Gastroenterology Maimonides Medical Center 4802 Tenth Avenue Brooklyn NY 11219 USA

## Abstract

The functional lifetime for biliary endoprostheses has typically been 7 months. When combined with sphincterotomy for common bile duct stones, it affords an alternative to surgery in high risk patients. Biliary endoprostheses often require replacement in these patients, though recent reports suggest they are functioning longer. We present an 85-year-old asymptomatic woman with a 6-year-old biliary endoprosthesis, believed to be the longest functioning stent reported in the literature.


*Diagnostic and Therapeutic Endoscopy*, Vol. 1, pp. 113–115
Reprints available directly from the publisher
Photocopying permitted by license only

© 1994 Harwood Academic Publishers GmbH
Printed in Malaysia

### A Biliary Endoprosthesis Functioning After Six Years

ALAN PLUMER[1], K. ISWARA[2] and JEROME H. SIEGEL[3]

[1]*Department of Medicine, Division of Gastroenterology, Maimonides Medical Center, Brooklyn, NY;* [2]*Maimonides Medical Center, Brooklyn, NY;* [3]*Beth Israel Medical Center, North Division, New York, NY*

*(Received January 6, 1994; in final form February 10, 1994)*

The functional lifetime for biliary endoprostheses has typically been 7 months. When combined with sphincterotomy for common bile duct stones, it affords an alternative to surgery in high risk patients. Biliary endoprostheses often require replacement in these patients, though recent reports suggest they are functioning longer. We present an 85-year-old asymptomatic woman with a 6-year-old biliary endoprosthesis, believed to be the longest functioning stent reported in the literature.

**KEY WORDS:** biliary stent, endoprosthesis, endoscopic retrograde cholangiopancreatography, functional survival

### INTRODUCTION

Endoscopic sphincterotomy (ES) with placement of a biliary endoprosthesis for the management of common bile duct (CBD) stones, initially described in 1982, is now becoming routine (Cotton and Williams, 1982). The usual indication is for patients unfit for surgery with unextractable CBD stones. Typically, patients are elderly with a debilitating disease and short life expectancy. The goal of therapy is to prevent recurrence of cholestasis and cholangitis. Success is achieved in approximately 90% of cases, with a mortality rate of 1%.

In comparison, surgical management may result in a 10–20% perioperative mortality (Soomers *et al.*, 1990). The median functional survival of a prosthesis is 32 weeks. Some prostheses have remained functional for as long as 5 years (Nordback, 1989). We report a prosthesis functioning for more than 6 years. This case further demonstrates the usefulness of ES with a biliary endoprosthesis in the long-term management of retained bile duct stones.

### CASE REPORT

An 85-year-old woman was referred for evaluation of a 10-lbs. weight loss, elevated liver tests, and an abnormal sonogram. Her main complaint was jaundice. She had no abdominal complaints. The patient's past history was significant for episodes of recurrent choledocholithiasis and cholecystitis. In 1986, an ERCP revealed moderate dilatation of the CBD and multiple calculi measuring up to 2.5 cm in diameter. Since she was not a candidate for surgery, sphincterotomy with stone extraction was planned. Stone entrapment was unsuccessful and a 10 Fr prosthesis was placed into the CBD for decompression. Three days later, ERCP was repeated and the stones were successfully crushed, fragmented, and removed using a lithotripter basket. It was not certain whether all of the stone fragments were removed, and the prosthesis was left in place to avoid cholangitis. She was closely followed and ERCP was not repeated until this admission.

On her current examination, she was a thin jaundiced afebrile woman, whose exam was remarkable only for right upper quadrant abdominal tenderness. Serum alkaline phosphatase was 400 mg/dl, total bilirubin was 2.8 mg/dl, SGOT (AST) was 120 mg/dl and SGPT (ALT) was 138 mg/dl. After phrophylactic antibiotics were given, a repeat ERCP was performed (Fig. 1). The original 6-year-old prosthesis was removed (Fig. 2) and replaced with two 7 Fr prostheses. The original stent was obstructed, calcified, and had a bilious coat. A 3-cm stone was demonstrated by ERCP but no attempt was made to remove the stone. The patient has continued to do well, without complication.

Address for correspondence: Alan Plumer, M.D., Dept. of Gastroenterology, Maimonides Medical Center, 4802 Tenth Avenue, Brooklyn, NY 11219.

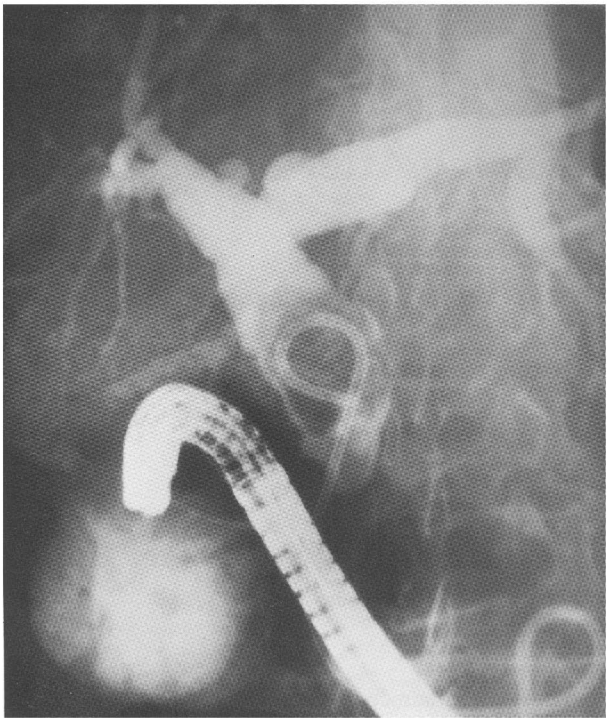

**Figure 1** ERCP showing a 10 French pigtail catheter in the common bile duct. The large common bile duct stone can also be seen.

### DISCUSSION

When patients unfit for surgery undergo ES, and stone extraction is unsuccessful, a biliary endoprosthesis should be placed to allow for adequate drainage (Siegel and Yatto, 1984). While the prosthesis may provide a lumen for drainage, more importantly it prevents stone migration and impaction, thereby permitting bile flow. A pigtail catheter is preferred because it is more resistant to displacement due to its configuration (Cotton and Williams, 1982; Oh *et al.*, 1992).

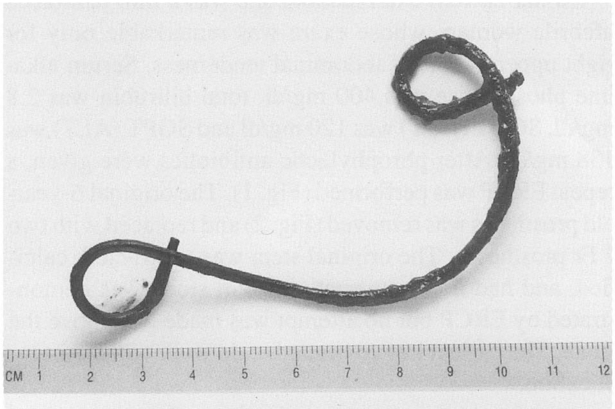

**Figure 2** The 10 French pigtail catheter after removal. Note the calcified film surrounding the prosthesis.

The median survival time for biliary endoprosthesis function is greater for 10 Fr than 7 Fr prostheses, and has been reported to be 32 weeks. Occlusion results from accumulation of bacterial biofilm and smaller stents occlude earlier (Nordback, 1989, Gholson and Burton, 1991). Until recently, ES with biliary endoprosthesis placement was used for both acute and short-term management in patients with retained CBD stones. Patients with short life expectancies requiring palliation, were ideal candidates since the function of the prosthesis would outlast the patient. We believe that the 6-year survival in this case is the longest documented. Other studies have reported stent survival up to 5 years and recommend that long-term management may be a realistic consideration (Nordback, 1989). This case supports that belief. Furthermore, the idea that the prosthesis' ability to prevent stone migration and impaction is more important that patency of the prosthesis itself is supported by the fact that the original 10 Fr prosthesis was clogged and calcified. Other investigators have found similarly that the prosthesis' lumen may become clogged within months, however adequate biliary flow is maintained (Cotton and Williams, 1982; Siegel 1992).

Several studies report successful clearance of the CBD in 80–95% of patients with ES alone (Davidson *et al.*, 1988; Vaira *et al.*, 1989). If biliary drainage is not attempted when the CBD cannot be cleared, the complication rate approaches 30% (Davidson *et al.*, 1988; Vaira *et al.*, 1989; Ingoldby *et al.*, 1989). Vaira *et al.* showed that complication rates are significantly reduced (9.9%) by placing either a naso-biliary drain or a pig-tail stent. In 71% of their patients with retained CBD stones, pig-tail stents were successfully used for indefinite management (Vaira *et al.*, 1989).

When CBD clearance cannot be achieved by ES, and surgery is not an option, a biliary endoprosthesis should be placed. Although occlusion commonly occurs, regular stent exchanges are not necessary until symptoms develop, which can be as long as 6 years.

### ACKNOWLEDGEMENTS

We would like to thank and acknowledge Lillian Rosado and Willie Wood for their fine work and dedication.

### REFERENCES

Cotton P. B., Williams C. B. (1982) Big stones. In: *Practical Gastrointestinal Endoscopy*, 2nd Edition: Boston, Blackwell.

Davidson B. R., Neoptolemos J. P., Carr-Locke D. L. (1988) Endoscopic sphincterotomy for common bile duct calculi in patients with gallbladder in situ considered unfit for surgery. *Gut*, 29:114–120.

Gholson C., Burton F. (1990) Obstructive jaundice. *Postgrad Med*, 90:107–116.

Ingoldby C. J. H., Et-Saadi J., Hall R. I., Denyer M. E. (1989) Late results of endoscopic sphincterotomy for bile duct stones in elderly patients with gallbladders in situ. *Gut*, 39:1129–1131.

Nordback I. (1989) Management of unextractable bile duct stones by endoscopic stenting. *Annales' Chirurgiae et Gynaecologiae*, 78:290–292.

OH D. J., Gilliam J. III, Zagoria R., Young G. (1992) Interventional endoscopy of the biliary and pancreatic ducts: current indications and methods. *AJR*, 158:243–250.

Siegel J. H. (1992) Endoscopic retrograde cholangio-pancreatography technique, diagnosis and therapy. New York, Raven.

Siegel J., Yatto R. (1984) Biliary endoprostheses for the management of retained common bile duct stones. *Am J Gastroenterol*, 79:50–54.

Soomers A. J., Nagengast F. M., Yap S. H. (1990) Endoscopic placement of biliary endoprostheses in patients with endoscopically unextractable common bile duct stones. *Endoscopy*, 22:24–26.

Vaira D., Ainley C., Williams S., *et al.* (1989) Endoscopic sphincterotomy in 1000 consecutive patients. *The Lancet*, August:431–433.

